# The Fireside

**Published:** 1864-12

**Authors:** 


### THE FIRESIDE.

Mr. Editor:—I like the name of your monthly. It stands as a protest against a certain innovation which should be exterminated, for we are in danger soon of having no firesides! It is the fashion up here in the country to build houses without fire-places, and to such an extent is this carried, that, in order to be fashionable, many have taken down the chimneys and removed their fire-places, just putting a chimney on the top! But what right has a house to a chimney on its top, if it have not a fire-place in its body? This is carrying false colors—it is telling a lie with brick and mortar! And how can there be a fireside without a fire-place? Do you say the stove or the furnace will supply the deficiency? Never! They are gloomy and cheerless in comparison with the great big fire-place and the roaring fire of the olden times! Besides, there is no ventilation in these country farm-houses without fire-places. There is a want of pure air—a superabundance of steam and bad breath! Hence the health must suffer. As editor of a health-journal, I hope you will protest against this innovation. The man who builds a house without a fire-place is mad, or has some evil design against his family, for he is dooming his household to miserable ill-health, as well as robbing them of the cheerful fireside, where parents and children, brothers and sisters, friends and kindred, may meet with thankfulness and joy, while the cheerful fire blazes on the hearth!

Alas! we have no hearths now! Hence, family ties are weakened, and that blissful word, HOME, awakens not the joy that once it did? I speak of the country, and if your readers would see a right old-fashioned fire-place, and a roaring fire after the old-time fashion, let them visit my father's kitchen! There he may see them; for my mother would as soon tolerate a locomotive in her kitchen as a stove, and a house without a fire-place would be deemed by her unfit for any thing but a barn, and in this opinion I think she would be right!

If your JOURNAL OF HEALTH will save to us some of our firesides, and make them cheerful and happy, I shall be glad. Your readers, however, must remember that TO BE HAPPY THEY MUST BE GOOD.

